# Theobald Palm and His Remarkable Observation: How the Sunshine Vitamin Came to Be Recognized

**DOI:** 10.3390/nu4010042

**Published:** 2012-01-17

**Authors:** Russell W. Chesney

**Affiliations:** The Department of Pediatrics, Le Bonheur Children’s Hospital, Children’s Foundation Research Center, The University of Tennessee Health Science Center, Memphis, TN 38103, USA; Email: rchesney@uthsc.edu; Tel.: +1-901-287-6106; Fax: +1-901-287-5028.

**Keywords:** vitamin D, nutritional rickets, photobiology, sunlight, UVB wavelength rays

## Abstract

The seminal discovery that sunlight was important in the prevention of nutritional rickets was made in 1890 by Theobald A. Palm, a medical missionary who contrasted the prevalence of rickets in northern European urban areas with similar areas in Japan and other tropical countries. He surmised that exposure to sunlight prevented rickets. Over the next 40 years his observation led to an understanding of ultraviolet irradiation and its role in vitamin D synthesis. This opened a new era of appreciation for the curative powers of the sun and “the sunshine vitamin”. While Palm’s observations were in some ways obscure, they had a potent effect on the development of photobiology.

## 1. Introduction

The prototypic disorder of vitamin D-deficiency in growing children is rickets. In nearly epidemic proportions in the west and south of England in the early 1600s, rickets (known as “the English disease”) was soon evident in numerous parts of northern Europe and North America [1,2,3]. Because its prevalence was as high as 60–80% in children living in poverty, many early pediatricians sought its cause. Theories of its etiology included an infectious cause, a congenital condition, the result of confinement, or bowel flora autocontamination [3,4]. The various schools of thought converged by the early 1920s, and people realized that rickets was a result of lack of an anti-rachitic nutrient obtained in the diet or by exposure of the skin to ultraviolet radiation [2,5]. A nutritional basis for rickets resonated with the discovery of “vital substances”, or “vitamines” (now vitamins), found lacking in the diets of victims of scurvy, beri-beri and pellagra between 1880 and 1915. In contrast, rickets could also be prevented or cured by sunshine. What observations led to recognition of the power of sunlight and the notion of a “sunshine vitamin”?

At present we recognize that the bone undermineralization disorder of childhood called rickets is a vitamin D-deficiency disorder [6]. We also appreciate that, in contrast to most other vitamins, vitamin D is not really a vitamin (a substance required for life), but rather a prohormone of the vitamin D-parathyroid hormone-calcium feedback system [7,8]. The capacity to convert 7-dehydrocholesterol in keratinocytes into vitamin D (calciferol) depends on exposure to ultraviolet B rays from sunlight or artificial sources. This article examines the elucidation of the capacity of the sun to both prevent and treat rickets, a condition of growing children obviating normal mineralization of the growth plates of long bones. A central figure, Theobald A. Palm, M.D., made the seminal discovery in 1890 of the anti-rachitic properties of sunshine. The discovery went essentially unremarked until World War I, but when it was finally recognized it ushered in a long, pro-sunshine era.

## 2. History

To appreciate the insight of Theobald Palm, we must return to the 1800s. The search for the etiology of and the ultimate cure for childhood rickets was a passionate quest. “Rickets is a disease of civilization, and is so frequently found in the large cities of America and Europe that it is doubtful whether the children of the poorer classes ever wholly escape it” [9]. Urban prevalence figures ranged from 60 to 90 percent in the late 19th and early 20th centuries [3]. An extensive literature concerning rickets led Zappert to state that it had become “an actual battle-ground for specialists in children’s diseases” [3]. As of 1910, the etiology of rickets was still puzzling [2,3,4,10] but by 1920, the presence of an “anti-rachitic factor” in fats, especially cod liver oil, and the role of UVB wavelength rays in curing or preventing the disorder, were established in a preliminary fashion [9,11,12,13]. By 1930 vitamin D had been discovered in cod liver oil by McCollum and the sunshine era was fully established. Cod liver oil was referred to as “sunshine in a bottle” to be employed if sunlight was not available [14]. What had happened and how was sunlight considered a cure?

After finishing the Edinburgh University School of Medicine, Theobald Adrian Palm, MA, MD, the son and grandson of Scottish Presbyterian missionaries, selected the Edinburgh Medical Mission. When he finished school, his home was listed as Ceylon, where he had been born in Colombo some 25 years earlier. He spent 10 years in Japan, moving to Niigata, a treaty port, in 1875 [15]. While in Japan, Palm noted that rickets was essentially absent, in contrast to the situation in the United Kingdom (UK). He first wrote on the “want of light” in a letter [16] to the *British Medical Journal* in 1888, when he was living in Birkenhead, near Liverpool, and saw children with rickets. Palm speculated that the therapy of rickets should include “the systematic use of sun-baths”.

Theobald Palm wrote in more detail about his observations after collecting information with a 3-pronged approach. First, he wrote to and assembled the replies from medical missionaries in the southeast region of Asia and North Africa. Second, he analyzed the topography of rickets in the UK based upon a medical research report [17]. Third, he catalogued rickets rates in other parts of Europe.

**Figure 1:**
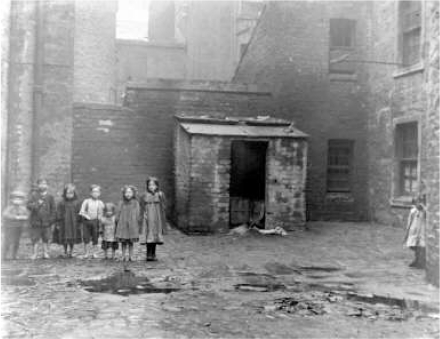
Typical living conditions in the Gorbals in 1912. This region of Glasgow was the most notorious slum in the United Kingdom. Reproduced with permission from [20], Copyright © The Mitchell Library, Glasgow Life.

Other medical missionaries from China, Mongolia, India, Morocco, Ceylon, and other parts of Japan rarely or never encountered rickets [18]. The geography of rickets appeared to involve the temperate latitudes of Europe: Germany, England, Holland, Belgium, France and northern Italy; Southern Italy, southern Spain, Turkey and Greece “enjoy a notable immunity from it” [18]. Rickets abounded in the UK in large towns and industrialized regions: Glasgow and Edinburgh and the coal-bearing regions of the Clyde-Forth region and five great regions of England and Wales. These included the Tyne area, Lancaster and Yorkshire, Birmingham and Manchester, Cardiff and Swansea, and the whole of London except for prosperous areas. These, apart from London, were the coal-mining districts of Britain, and London was the center of the distribution of coal [19]. The area between Glasgow and Edinburgh, known as the Clyde River–Forth Estuary Valley, abounded with coal, coke, and iron. It was a region of iron and steel works and of the manufacture of bridges, steam engines and ships. Both cities were hazy and smoggy, and the air was filled with soot (Figure 1). Edinburgh was referred to as “auld reeky”. It was also an area with the highest prevalence of rickets [4].

Based upon these observations, Palm was struck by the fact that children in tropical zones were exposed to filth, inadequate sanitation, unsafe water and dysentery, yet they were free of rickets. These children in Japan, China, India and other sites had other common diseases seen in rachitic children, namely pneumonia, scrofula and tuberculosis (TB) [21]. Hence, the known infectious comorbidities found in rachitic children could prevail in a population where rickets was not encountered [18].

Had Palm remained a medical missionary he might never have made the observations he did regarding rickets and sunshine. However, in 1884 he returned to practice in England; specifically Wigton, Cumberland in the northwest of England, not far from Carlisle and west of the Tyne coal-mining district [15]. There, Palm encountered rachitic children, and he began his inquiries. He noticed, from the epidemiologic map of the prevalence of rickets published by the Collective Investigation Committee of the British Medical Association in 1889, that the disease was common in “large towns and thickly peopled districts” [18]. He had spent a year in Tokyo and never encountered rickets, nor had he seen it in the smaller city of Niigata. He remarked on the universality of rickets in Glasgow, and its scarcity in the Highlands, which was even farther to the north in Scotland.

## 3. Results

Palm could only reconcile these findings with the lack or adequacy of sunlight. Others noted the predominance of rickets in urban areas. Both August Hirsch in Germany and the Investigation Committee of the British Medical Association created maps of rickets-prone zones, but they chose to emphasize crowding, air quality and even the soil [15]. Palm focused upon the sun. He recommended scientific observation of the effects of sunlight on health, the use of sunbaths and the relocation of rachitic children to areas where sunshine is common [18]. There had only been one previous mention of sunlight’s role in the cure of rickets, by Jedrzej Sniadecki in 1822, who noted less rickets in children from rural districts of western Poland [22], but this was a local finding. Sniadecki wrote about rachitic children in the densely populated, narrow and dark city streets, but the accuracy of his observation was only recognized a century later. Mozolowski quotes Sniadecki in his 1822 book *On the Physical Education of Children* as saying that the direct action of the sun was important in the cure and prevention of rickets [22]. Theobald Palm was able to use global surveys from Europe and the UK to gain a broader picture and publish an extensive report in the *Practitioner* [18].

**Figure 2:**
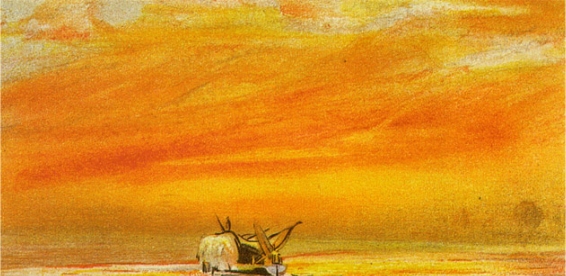
Sunset by British artist William Ashcroft depicting the colorful skies after the eruption of Krakatoa in 1883. Reproduced with permission from [24], Copyright © 2012 Photo Researchers, Inc.

Palm’s perceptions may have been influenced by the increased atmospheric dimness due to the 1883 eruption of Krakatoa [23]. Ash rose to a height of 80 km. Average global temperatures fell by more than two degrees the next year and weather was erratic for several years, until at least 1889. Unusual amounts of sulfur dioxide were also released into the stratosphere and were carried by wind all over the earth. This SO_2_ also led to enhanced cloud reflectivity (albedo), which reflected more sunrays away from the earth. Sunsets were spectacular. There was a Bishop’s Ring around the sun by day and a purple light at twilight. The British artist William Ashcroft depicted these red sunsets in thousands of color sketches, which became his trademark (Figure 2).

Palm’s observations were largely ignored, but in 1904 Buchholz reported the curing of 16 rachitic children with the rays of “glühlicht” [25]. “Glühlicht” may be translated as “glow” light, or incandescent light. Jan Raczynski in 1912 noted the seasonal incidence of rickets with new cases appearing in his clinic between January and May and decreasing in June through December [26]. Raczynski went one step further, exposing one of two newborn pups to sunlight and leaving the other in the dark. The light-exposed pup was free from rickets but not the other [26].

Rickets was the most common cause of deformity in the 17th to early 20th centuries [27]. As a chronic condition, it attracted the attention of sanitarium enthusiasts. Both Florence Nightingale and Hugh Owen Thomas advocated cleanliness, fresh air and sunlight. Thomas was also an Edinburgh graduate and a solar light enthusiast; in 1878 he actually treated rachitic children with the abundant sunlight on the balconies of Sea Side Hospital at Rhyl, Wales, focusing on the deformation of the spine associated with osseous tuberculosis [28]. This therapeutic approach was consistent with Palm’s admonition that rachitic children be relocated “as early as possible from large towns to a locality where sunshine abounds and the air is dry and bracing” [18].

While rickets is the osseous form of severe vitamin D deficiency and nutritional rickets is the most common form, there are other types of hypocalcemic and hypophosphatemic rickets, frequently with a hereditary basis and involving renal tubular transport or various aspects of vitamin D metabolism or receptor activity [29]. The discovery of the sun as a source of vitamin D did not end the epidemic of nutritional rickets. This crippling condition was ultimately cured or prevented after the classic studies of Edward Mellanby [30], the discovery of vitamin D by Elmer McCollum and his team [31], and supplementation of foodstuffs with vitamin D_2_ (ergocalciferol) by Harry Steenbock [32] and independently by Alfred Hess in the 1920s [33]. At the same time as landmark studies of the effects of cod lever oil administration by McCollum’s team, Hess’s and Steenbock’s work with animals, and Harriette Chick’s examination of rachitic Viennese orphans, the dual roles of diet and exposure to sunshine in the production of vitamin D were being recognized [34,35].

**Figure 3:**
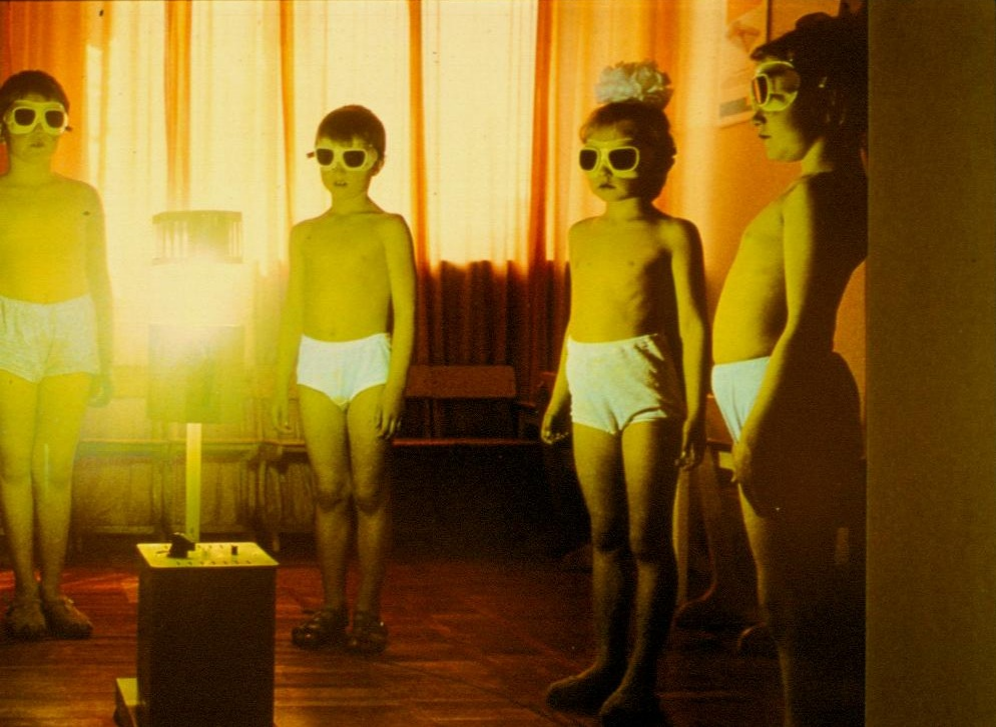
Irradiation of German children with quartz-mercury vapor lamps. It was recognized that the eyes should be protected from UVB rays.

During World War I, the German and Austro-Hungarian empires were under a stiff blockade imposed by the British navy, and food for civilians was totally inadequate. Rickets became even more prevalent in children in wartime Berlin and Vienna; even older children and young adolescents developed rickets [2,34,36], probably because of malnutrition and delayed onset of puberty. Many of these children were not only malnourished, but also were orphans. Orphanages were often disorganized and the children only got out of doors for limited amounts of time [34]. Kurt Huldschinsky, a Berlin pediatrician, noted the pale skin of his patients. He provided them with calcium supplements and irradiated them with quartz mercury-vapor lamps (Figure 3), which emit wavelengths ranging from 200 to 600 nm (UVB wavelengths are 290–320 nm), and then announced a cure [37,38]. This cure for rickets was rapidly confirmed in 100 other children [39]. Investigations on the severity of rickets and its response to therapy were greatly aided by the use of X-rays [2,40,41]. From serial views of limb X-ray studies, Huldschinsky demonstrated that light shined on only one arm cured rickets in both arms. He theorized that a chemical was synthesized in response to UV light that could diffuse throughout the affected child [37].

A New York pediatrician, Alfred Fabian Hess, observed that rickets was prevalent in immigrant (Italian) and African-American children and employed both sunlight and quartz crystal mercury-vapor lamps to prevent and heal rickets [42]. Rickets could be prevented by UVB wavelength rays (289 and 302 nm), but not by UVA wavelengths, above 320 nm [5].

The mechanism of exposure to UVB wavelength rays on rickets was then explored in a series of clever animal studies, including work in the Hess laboratory [5,43]. Initial studies seemed to show that the air of irradiated rat cages of rats fed a fat-free diet might hold the curative factor [44]. The investigators did not, however, remove the sawdust from the cages at the time of irradiation. Harry Steenbock and his group in Madison, Wisconsin demonstrated that the irradiated rats could transfer the curative factor if non-irradiated rats ate the excreta in the sawdust of the cage, or if they licked the oils from the fur of the irradiated rats [32,45,46,47]. Hess fed irradiated human cadaver skin to rats otherwise deprived of dietary fat and light, and prevented rickets [5]. He then collaborated with Adolph Windaus, a German cholesterol maven and future Nobelist, to discern the structure of the fat-soluble vitamin D, showing it was an activated ergosterol product [48]. Hess and Steenbock independently irradiated ergosterol and the green foodstuffs given rats, which prevented rickets despite a rachitogenic diet [43,46]. Steenbock patented the irradiation process and was able to develop a technique by which ergocalciferol was added to milk and other dairy products [49]. The supplement was set at 400 IU (10 μg) per liter, the amount found in a teaspoonful of cod liver oil of the time [50].

## 4. Discussion

Ultimately, from Palm’s powerful observations concerning the importance of light, came the sunshine movement. This era was punctuated by newsreels of healthy-appearing men and women exercising in bright sunlight. Key was the understanding of the value of UVB wavelength rays in both the prevention and cure of rickets, as well as the role of latitude in determining the amount of sunshine that could reach the skin in various seasons. Mothers were urged to give their babies a tan, and, failing this, to use “bottled sunshine”, namely, cod liver oil [14]. Although the peak of the sunshine movement was some 60 to 90 years ago, the rising rate of skin cancer and recognition of the DNA damage inherent in sunburn has led to current, strict guidelines [51,52]. Today we are strongly advised to limit sun exposure, wear more clothes and sunglasses, seek shade and use high SPF sunscreens. Dietary supplementation rather than sunlight is recommended to provide vitamin D.

## 5. Conclusions

In conclusion, Theobald Palm believed in “sunshine as a means of health” [18]. He urged the “abatement of smoke” and the “multiplication of open spaces, especially as play-grounds for the children of the poor” [18]. Writing in 1890, his analysis of the geographic distribution of rickets and recognition of the power of sunlight led to a seminal observation that led to subsequent studies in the early 20th century that expanded upon and proved his theories.
